# Body composition changes at 12 months following different surgical weight loss interventions in adults with obesity: A systematic review and meta‐analysis of randomized control trials

**DOI:** 10.1111/obr.13442

**Published:** 2022-03-08

**Authors:** Amy Sylivris, Jakub Mesinovic, David Scott, Paul Jansons

**Affiliations:** ^1^ Department of Medicine, School of Clinical Sciences at Monash Health Monash University Clayton Victoria Australia; ^2^ Institute for Physical Activity and Nutrition (IPAN), School of Exercise and Nutrition Sciences Deakin University Geelong Victoria Australia

**Keywords:** bariatric surgery, body composition, obesity

## Abstract

To determine relative lean mass and fat mass changes in adults with obesity following surgical weight loss interventions, a systematic review and meta‐analysis was conducted. The Cochrane Central Register of Controlled Trials, PubMed, Web of Science, EMBASE, and Scopus were screened for eligible studies. Inclusion criteria included randomized controlled trials (RCTs) performed in populations with obesity (body mass index ≥30 kg/m^2^) aged over 18 years, who underwent any type of bariatric surgery and reported body composition measures via dual‐energy X‐ray absorptiometry or bio‐electrical impedance analysis. Authors conducted full text screening and determined that there were six RCTs eligible for inclusion, with data extracted at 12 months post‐surgery. Meta‐analysis revealed that, relative to gastric banding, Roux‐en‐Y gastric bypass (RYGB) led to greater total body mass loss (mean difference [MD]: −9.33 kg [95% CI: −12.10, −6.56]) and greater fat mass loss (MD: −8.86 kg [95% CI: −11.80, −5.93], but similar lean mass loss (MD: −0.55 kg [95% CI: −3.82, 2.71]. RYGB also led to similar changes in total body mass, fat mass, and lean mass compared with sleeve gastrectomy. RYGB results in greater 12‐month weight and fat loss, but similar changes in lean mass, compared with gastric banding. Further RCTs comparing body composition changes following different bariatric surgery procedures are required.

## 1 INTRODUCTION

By 2030, nearly 60% of the global population is projected to be afflicted with obesity.^1^ With health sequelae such as hypertension, stroke, cardiovascular disease, type 2 diabetes mellitus (T2DM), obstructive sleep apnea, osteoarthritis, and liver disease, obesity is one of the most significant health challenges of the modern era.^2^

Bariatric surgery elicits weight loss by either restricting calorie intake or absorption and often results in long‐term weight loss (50–60% of excess weight loss is sustained at 10 years following surgery), remission of T2DM, and reduced mortality.^3^ Furthermore, bariatric surgery decreases chronic disease risk and medication dosages to manage associated conditions such as pain and respiratory, cardiovascular, diabetes‐related, and gastroenterological complications.^4^

There are several types of bariatric surgery, and the most commonly performed are Roux‐en‐Y gastric bypass (RYGB), sleeve gastrectomy (SG), and gastric banding.^5^ RYGB involves creating a small pouch of stomach that is attached directly to the jejunum, which re‐routes ingested food directly from the esophagus to the jejunum.^6^ In SG, the normal intestinal pathway remains intact, but the size of the stomach is reduced by about 80% via surgical removal; and in gastric banding, a band is placed around the proximal stomach so that a small pouch is created proximal to the band, restricting the amount of food entering the region of the stomach distal to the band.^6^ The mechanism by which weight loss occurs varies between procedures, but typically involves restriction of food intake, as well as a change in hormonal and signaling pathways that affect appetite and metabolism.^6^

A disadvantage of successful weight loss interventions is that they often result in significant bone and muscle loss,^7^, ^8^ and this may lead to negative outcomes including increased fracture risk.^9^, ^10^ Maintenance of lean muscle mass is beneficial for metabolic health and decreases fracture risk.^11^, ^12^, ^13^ Evidence also suggests greater lean mass is protective against T2DM, osteoporosis, and obesity.^14^ In comparison, loss of lean mass is associated with declines in muscle strength^15^ and function^16^ and an increased risk for all‐cause mortality.^17^ In individuals undergoing weight loss, losses in lean mass also contributes towards subsequent weight regain.^18^ Therefore, interventions that maximize fat mass losses while retaining lean mass are likely to result in optimal outcomes for people afflicted with obesity.

A recent meta‐analysis^19^ that investigated body composition changes following bariatric surgery in non‐randomized studies found an average lean mass loss one‐year post‐RYGB of 10 kg (*P* < 0.001), but despite this loss, lean mass to body mass ratio increased by almost 12%. This meta‐analysis also reported smaller absolute lean mass losses post gastric banding (7 kg) and SG (9.5 kg), with smaller increases in lean mass to body mass ratios (8.1% and 5.7%, respectively).^19^ Therefore, RYGB might lead to greater fat mass losses while preserving lean mass relative to other surgical weight loss interventions. To date, no meta‐analyses have directly compared fat and lean mass changes following different types of bariatric surgeries in randomized controlled trials (RCTs) to establish whether some lead to a more favorable body composition relative to others. Thus, the aim of this systematic review and meta‐analysis was to compare changes in body composition (in particular, fat mass and lean mass changes) in adults afflicted with obesity undergoing different surgical weight loss interventions commonly utilized worldwide.

## 2 METHODS

This study was conducted in accordance with the 2020 Preferred Reporting Items for Systematic Reviews and Meta‐Analysis (PRISMA) statement,^20^ and a PRISMA checklist can be found in Table S1. The study protocol was registered with the International Prospective Register of Systematic Reviews (PROSPERO) (registration no: CRD 42020173485). A protocol was not prepared for this systematic review and meta‐analysis.

### 2.1 Search strategy and study selection

We systematically searched Embase (1980 to Present), PubMed, Web of Science, Scopus, and Cochrane Central Register of Controlled Trials for relevant articles from inception until September 2021. We also searched bibliographies of included articles and our personal reference libraries. Our complete search strategy can be found in Table S2. Title, abstract, and full‐text screening was performed by two independent reviewers (AS and PJ) using Covidence software (Veritas Health Innovation, Melbourne, Australia).

We included parallel‐group RCTs with populations aged 18 years and over afflicted with obesity (body mass index [BMI] > 30 kg/m^2^) and underwent any type of bariatric surgery procedure and reported body composition measures in the form of fat or lean mass via dual‐energy X‐ray absorptiometry (DXA) or bio‐electrical impedance analysis (BIA). Studies were excluded if they were not published in English.

### 2.2 Data extraction and quality assessment

Data were independently extracted by two reviewers (AS and PJ). We extracted health status, age, sample size, age, post‐surgery fat mass, fat‐free mass, body mass, and lean mass data from eligible studies at baseline and at 12 months post‐surgery. For data extraction and analysis, we combined adjustable gastric banding with non‐adjustable gastric banding into one group, as these two procedures are comparable in terms of weight loss mechanism and overall efficacy.^21^

Fat‐free mass and lean mass are composed of different body composition compartments, with the key difference being the addition of bone mass in fat‐free mass measurements. However, despite fat‐free mass and lean mass being different absolute values, the pre‐ and post‐surgery change in these body composition compartments is comparable. Thus, change in fat‐free mass was considered the same as change in lean mass for the analysis. We performed an additional sensitivity analysis to evaluate whether combining fat‐free mass and lean mass would yield similar results.

All calculations were performed using Review Manager (RevMan).^22^ Quality assessment (risk of bias) of included studies was performed by two reviewers (AS and PJ) using the Cochrane Risk of Bias 2 tool, and discrepancies were adjudicated by a third reviewer (JM).^23^ Studies were classified as “low,” “some concerns,” or “high risk of bias” based on the following five bias domains: (1) randomization process, (2) deviation from intended intervention, (3) missing outcome data, (4) measurement of the outcome, and (5) selection of the reported results.

A Grading of Recommendations, Assessment, Development, and Evaluations (GRADE) assessment was performed to evaluate the quality of the outcomes used in the meta‐analysis.^24^ The use of five different domains (risk of bias, inconsistency, indirectness, imprecision, and publication bias) is assessed to determine the degree of confidence in the estimate of effect derived from the meta‐analysis. The quality of evidence is defined as high, moderate, low, or very low.

### 2.3 Statistical analysis

Mean differences in body composition changes between the two intervention groups were calculated as the mean change in control group minus the mean change in the intervention group, or as mean follow‐up values in the control group minus mean follow‐up values in the intervention group. For studies missing standard deviations of mean changes,^25^, ^26^, ^27^ we imputed a correlation co‐efficient of 0.7 and performed sensitivity analyses with co‐efficient of 0.5 and 0.9. Body composition data were pooled using random‐effects meta‐analyses, based on the DerSimonian and Laird model.^28^ Tau^2^, *Q*‐statistic, and *I* ^2^ values were used to measure heterogeneity between studies. Meta‐analyses were performed using RevMan.^22^ A *P* value < 0.05 was considered statistically significant.

## 3 RESULTS

### 3.1 Literature search and study characteristics

**FIGURE 1:**
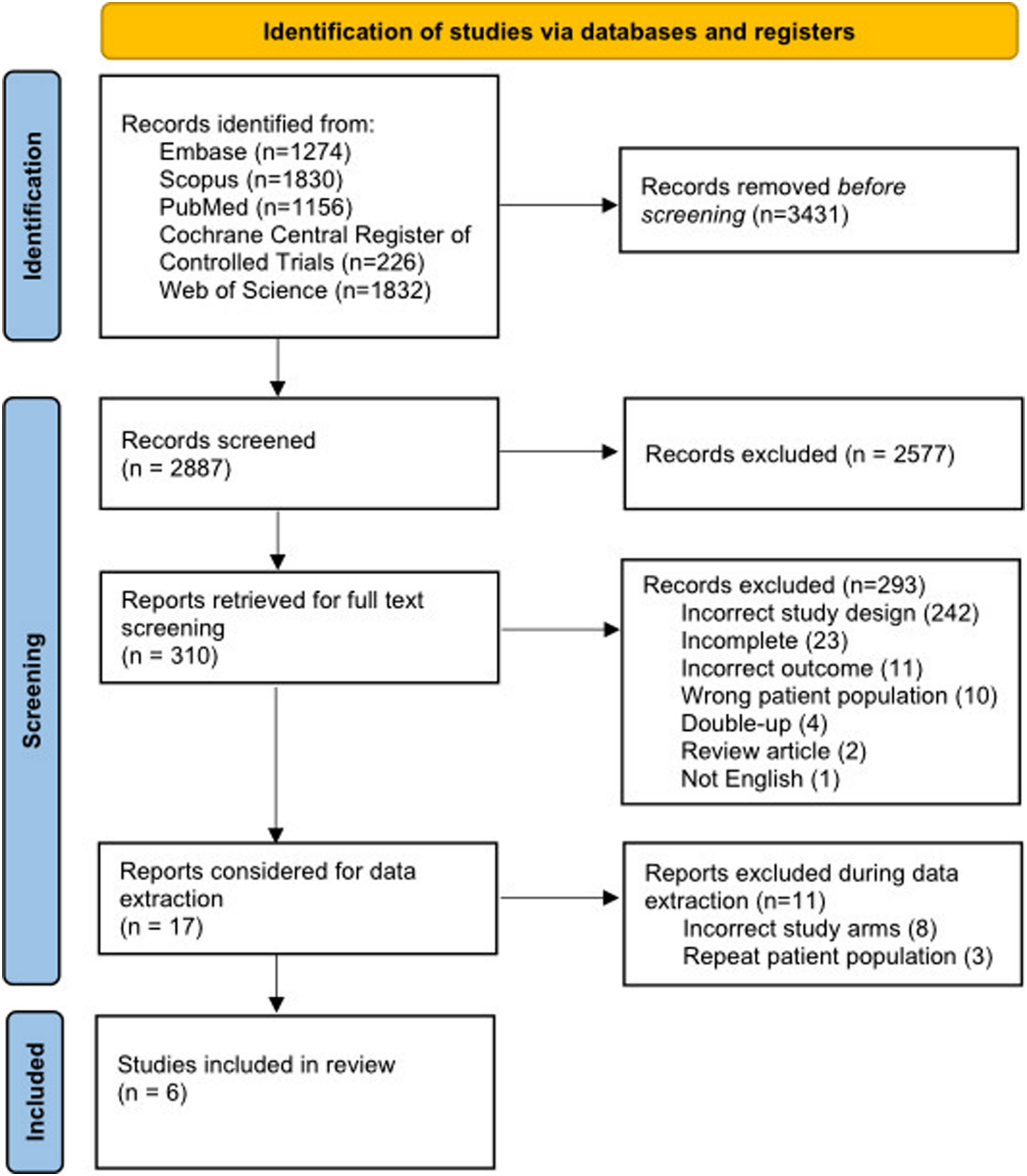
PRISMA diagram of information flow in this systematic review and meta‐analysis

Database searches identified 2887 unique citations. The full text of 310 articles were screened; data extraction was sought for 17 studies, but 11 were further excluded at this stage.^29^, ^30^, ^31^, ^32^, ^33^, ^34^, ^35^, ^36^, ^37^, ^38^, ^39^ Overall, six were included in the meta‐analysis (Figure 1). Descriptive characteristics of included studies are presented in Table 1. Four studies were conducted in individuals with T2DM,^7^, ^25^, ^26^, ^40^ and the other two studies included adults with obesity without specified health conditions.^27^, ^41^ Age varied across all studies (range: 18–65 years) and all had combined cohorts of men and women. All six studies included RYGB.^7^, ^25^, ^26^, ^27^, ^40^, ^41^ In two studies, RYGB was compared with gastric banding (either adjustable or vertical),^7^, ^41^ and in four studies, RYGB was compared with SG.^25^, ^26^, ^27^, ^40^ All studies provided data at exactly 12 months post‐surgery, however one study^7^ continued for a total of 36 months. Five studies included sufficient data to compare total body, lean, and fat mass changes.^7^, ^25^, ^26^, ^27^, ^40^ Of these five studies, two reported fat‐free mass instead of lean mass changes.^26^, ^40^ For one study,^41^ the total body mass changes were extracted from a graph using OriginLab.^42^ None of the eligible studies included protein supplementation. Body composition at baseline was similar in different bariatric surgery groups. In those who underwent RYBG, mean total body, fat, and lean mass at baseline were 114 kg, 49 kg, and 60 kg, respectively. Mean total body, fat, and lean mass at baseline were 112 kg, 50 kg, and 55 kg, respectively, in those who underwent gastric banding. In those who underwent SG, mean total body, fat, and lean mass at baseline were 115 kg, 47 kg, and 62 kg, respectively.

**TABLE 1:** Descriptive characteristics of randomized controlled trials comparing changes in body composition in adults with obesity undergoing weight loss while undertaking bariatric surgery

Study	Age, years	Sex	Population	Bariatric surgery	Body composition analysis	Initial body mass, kg (± SD)	Initial body fat, kg (± SD)	Number of participants	Mean weight change, kg (± SD)	Mean fat mass change, kg (± SD)	Mean lean mass change, kg (± SD)
Courcoulas 2015	25–55	M + W	T2DM, BMI 30–40	RYGB	GE/Lunar DXA	RYGB: 99.27 (12.69)	RYGB: 42.50 (7.50)	RYGB: 18	RYGB = −28.8 (5.04)	RYGB = −23.6 (3.9)	RYGB = −4.78 (2.28)
				LAGB		LAGB: 100.2 (13.68)	LAGB: 44.49 (7.46)	LAGB: 20	LAGB = −18.6 (5.66)	LAGB = −13.7 (4.33)	LAGB = −2.55 (2.43)
Guerrero‐Perez 2020	18–60	M + W	T2DM, BMI 35–43	RYGB	Hologic DXA	RYGB: 103.01	RYGB: 36.53 (8.09)	RYGB: 15	RYGB = −36.4 (8.33)	RYGB = −19.57 (5.78)	RYGB = −9.58 (7.79)
				SG		SG: 102.30	SG: 34.22 (5.57)	SG: 15	SG = −27.35 (8.07)	SG = −11.62 (4.22)	SG = −6.11 (6.27)
Keidar 2013	18–65	M + W	T2DM, BMI > 35	RYGB	Tanita BIA	RYGB: 118.04 (16.5)	RYGB: 49.6 (8.4)	RYGB: 19	RYGB = −30.24 (12.06)	RGYB = −23.9 (8.3)	RYGB = −5.3 (12.26)
				SG		SG: 117.9 (17.8)	SG: 51.4 (11.6)	SG: 18	SG = −33.8 (12.73)	SG = −24.9 (8.3)	SG = −9.3 (8.79)
Murphy 2018	22–55	M + W	T2DM, BMI 35–65	RYGB	GE/Lunar DXA	RYGB: 116 (22)	RYGB: 53.4 (15)	RYGB: 32	RYGB = −31 (30)	RYGB = −27 (19)	RYGB = −7.2 (18)
				SG		SG: 120 (25)	SG: 53.6 (15)	SG: 29	SG = −29 (29)	SG = −23 (16.9)	SG = −6.9 (22.8)
Olbers 2006	Mean 37.4	M + W	Not reported, BMI 35–50	RYGB	GE/Lunar DXA	RYGB: 123.2 (16.6)	RYGB: 54.1 (9.6)	RYGB: 29	RYGB = −36.56 (9.46)	RYGB = −26.9 (9.4)	RYGB = −4.4 (2.6)
				LVGB		LVGB: 123.3 (15)	LVGB: 56 (8.45)	LVGB: 31	LVGB = −28.95 (9.41)	LVGB = −20.2 (8.5)	LVGB = −5.5 (2.9)
Schneider 2016	18–64	M + W	Not reported, BMI > 35	RYGB	Hologic DXA	RYGB: 125.8 (22.7)	RYGB: 56.4 (12.1)	RYGB: 19	RYGB = −39 (17.35)	RYGB = −20.9 (14.33)	RYGB = −17.1 (9.65)
				SG		SG: 120.1 (19.2)	SG: 50.7 (8.6)	SG: 23	SG = −32.1 (13.73)	SG = −17.6 (7.66)	SG = −10.5 (9.16)

Abbreviations: BIA, bioelectric impedance analysis; BPDS, biliopancreatic diversion with duodenal switch; DEXA, dual‐energy X‐ray absorptiometry; LAGB, laparoscopic adjustable gastric banding; LVGB, laparoscopic vertical gastric banding; M, men; RYGB, Roux‐en‐Y gastric bypass; SD, standard deviation; SG, sleeve gastrectomy; T2DM, type 2 diabetes mellitus; W, women.

### 3.2 Risk of bias assessment

**FIGURE 2:**
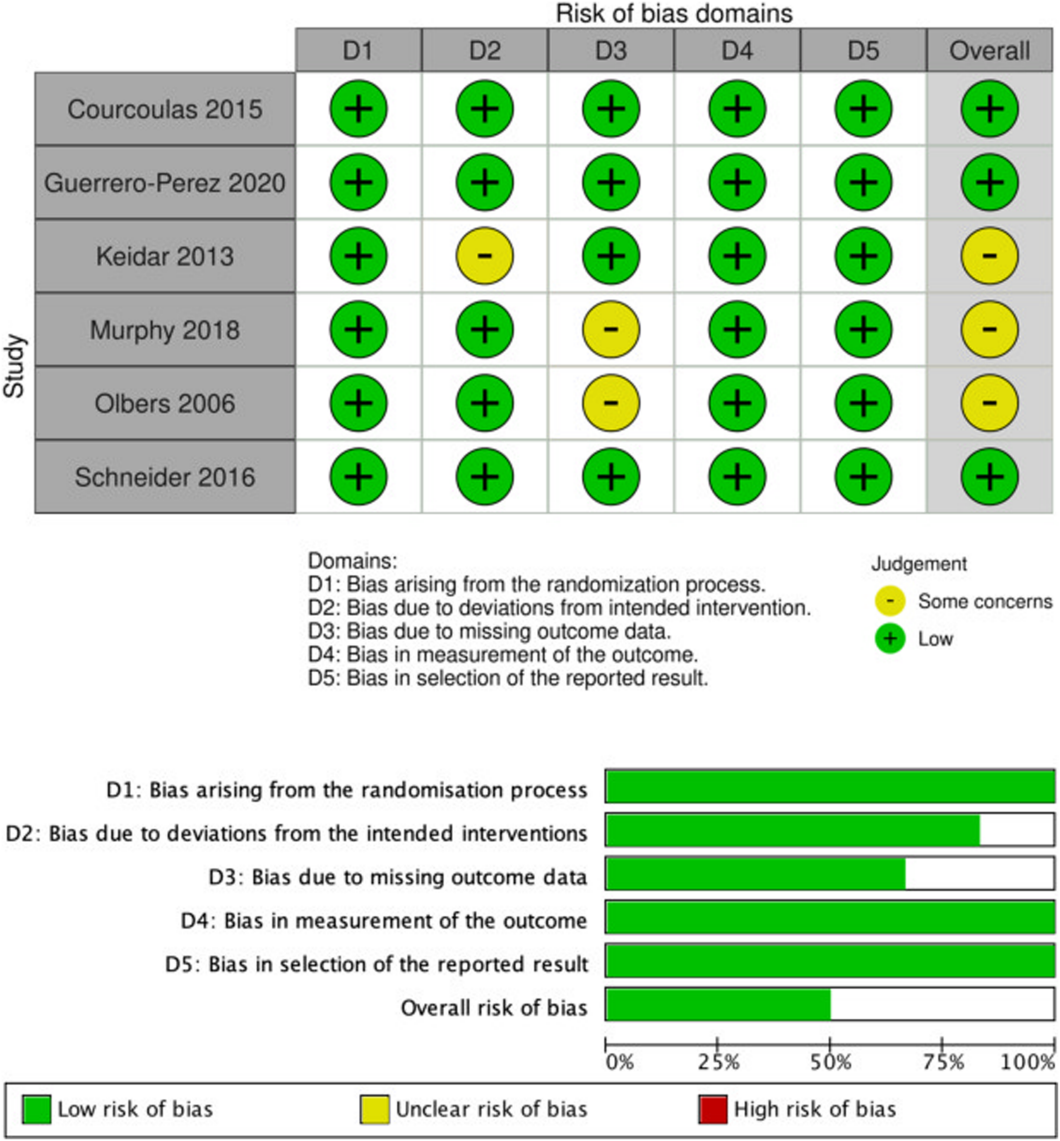
Study quality assessment using Cochrane Risk of Bias 2 tool

Study quality assessments performed using the Cochrane Risk of Bias 2 tool are presented in Figure 2. Most studies had low selection bias. One study^26^ was classified as having “some concerns” in domain 2 due to lack of concealment for treatment allocations. Two studies^40^, ^41^ were identified as having unclear risk of bias due to missing outcome data (domain 3). All other domains (1, 4, and 5) were considered low risk in all studies.

### 3.3 GRADE assessment

The GRADE assessment findings are summarized in Tables 2 and 3. For the RYGB versus gastric banding outcomes (Table 2), the quality of evidence was evaluated as either moderate or high. We downgraded the quality of evidence for lean mass outcomes due to substantial heterogeneity. Publication bias was undetectable for all outcomes due to the limited number of studies included. Otherwise, the outcomes were of high quality of evidence due to no serious risk of bias, no serious indirectness or imprecision for all outcomes, and a large effect size for the body mass and lean mass change outcomes.

**TABLE 2:** GRADE assessment for RYGB versus gastric banding

Outcome	Included studies	ROB	Inconsistency	Indirectness	Imprecision	Publication bias	Groups (RYGB/gastric banding)	Effect size (direction)	*P* value	95% CI	Quality
Body mass change	RCT^7^	No serious ROB	No serious inconsistency	No serious indirectness	No serious imprecision	Undetected^a^	47/51	−9.33 (RYGB)	<0.00001	(−13.79, −5.61)	High
Lean mass change	RCT^7^, ^41^	Moderate ROB	Serious inconsistency (substantial heterogeneity)	No serious indirectness	No serious imprecision	Undetected^a^	47/51	−7.42 (RYGB)	<0.0001	(−10.10, −4.74)	Moderate
Fat mass change	RCT^7^, ^41^	No serious ROB	No serious inconsistency	No serious indirectness	No serious imprecision	Undetected^a^	47/51	−0.83 (RYGB)	0.0003	(−4.65, 2.99)	High

^a^ Insufficient data to produce funnel plots.

**TABLE 3:** GRADE assessment for RYGB versus SG

Outcome	Included studies	ROB	Inconsistency	Indirectness	Imprecision	Publication bias	Groups (RYGB/SG)	Effect size (direction)	*P* value	95% CI	Quality
Body mass change	RCT^25^, ^26^, ^27^, ^40^	Serious ROB (significantly different results in the sensitivity analysis)	Serious inconsistency (substantial heterogeneity)	No serious indirectness	Serious imprecision (CI crosses line of no effect)	Undetected^a^	85/85	−4.04 (RYGB)	0.23	(−12.10, −6.56)	Very low
Lean mass change	RCT^25^, ^26^, ^27^, ^40^	Serious ROB (significantly different results in the sensitivity analysis)	Serious inconsistency (substantial heterogeneity)	No serious indirectness	Serious imprecision (CI crosses line of no effect)	Undetected^a^	85/85	−3.82 (RYGB)	0.11	(−8.52, 0.87)	Very low
Fat mass change	RCT^25^, ^26^, ^27^, ^40^	Serious ROB (significantly different results in the sensitivity analysis)	Serious inconsistency (substantial heterogeneity)	No serious indirectness	Serious imprecision (CI crosses line of no effect)	Undetected^a^	47/51	−2.11 (RYGB)	0.37	(−6.68, 2.46)	Very low

^a^ Insufficient data to produce funnel plots.

For the RYGB versus SG outcomes (Table 3), the quality of evidence was evaluated as very low for all. Again, publication bias was unable to be detected. Further, there was high risk of bias for all outcomes as the sensitivity analyses all yielded different results, as well and serious inconsistency due to heterogeneity of the results and serious imprecision due to wide confidence intervals.

### 3.4 RYGB versus gastric banding

**FIGURE 3:**
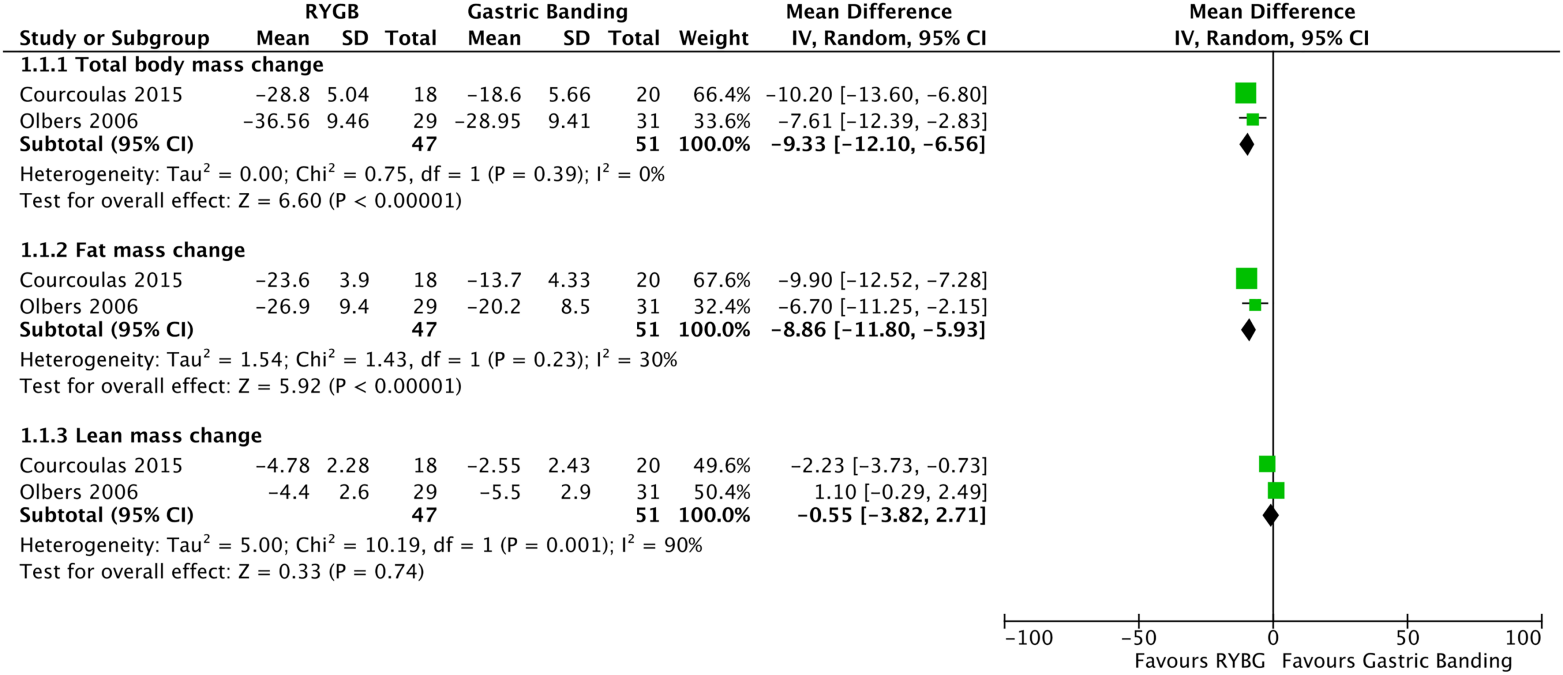
Mean differences in body composition outcomes after Roux‐en‐Y gastric bypass and gastric banding

Two studies were eligible for inclusion in the RYGB vs gastric banding meta‐analysis, with a total of 98 participants (Figure 3).^7^, ^41^ RYGB led to greater total body mass changes (mean difference [MD]: −9.33 kg [95% CI: −12.10, −6.56], *P* < 0.00001), fat mass losses (MD: −8.86 kg [95% CI: −11.80, −5.93], *P* < 0.00001), but similar lean mass losses (MD: −0.55 kg [95% CI: −3.82, 2.71], *P* = 0.74) compared with gastric banding. Heterogeneity was low in our fat mass analysis (Tau^2^ = 1.54, *I* ^2^ = 30%, and *Q*‐statistic = 1.43), but high in our lean mass analysis (Tau^2^ = 5.00, *I* ^2^ = 90%, and *Q*‐statistic = 10.19).

Of the studies, one^41^ was identified as having some concerns of risk of bias. Thus, there was only one study^7^ remaining for the sensitivity analysis. Alone, this study demonstrated that both fat mass and lean mass losses were significant at 12 months (Figure 3).

### 3.5 RYGB versus SG

**FIGURE 4:**
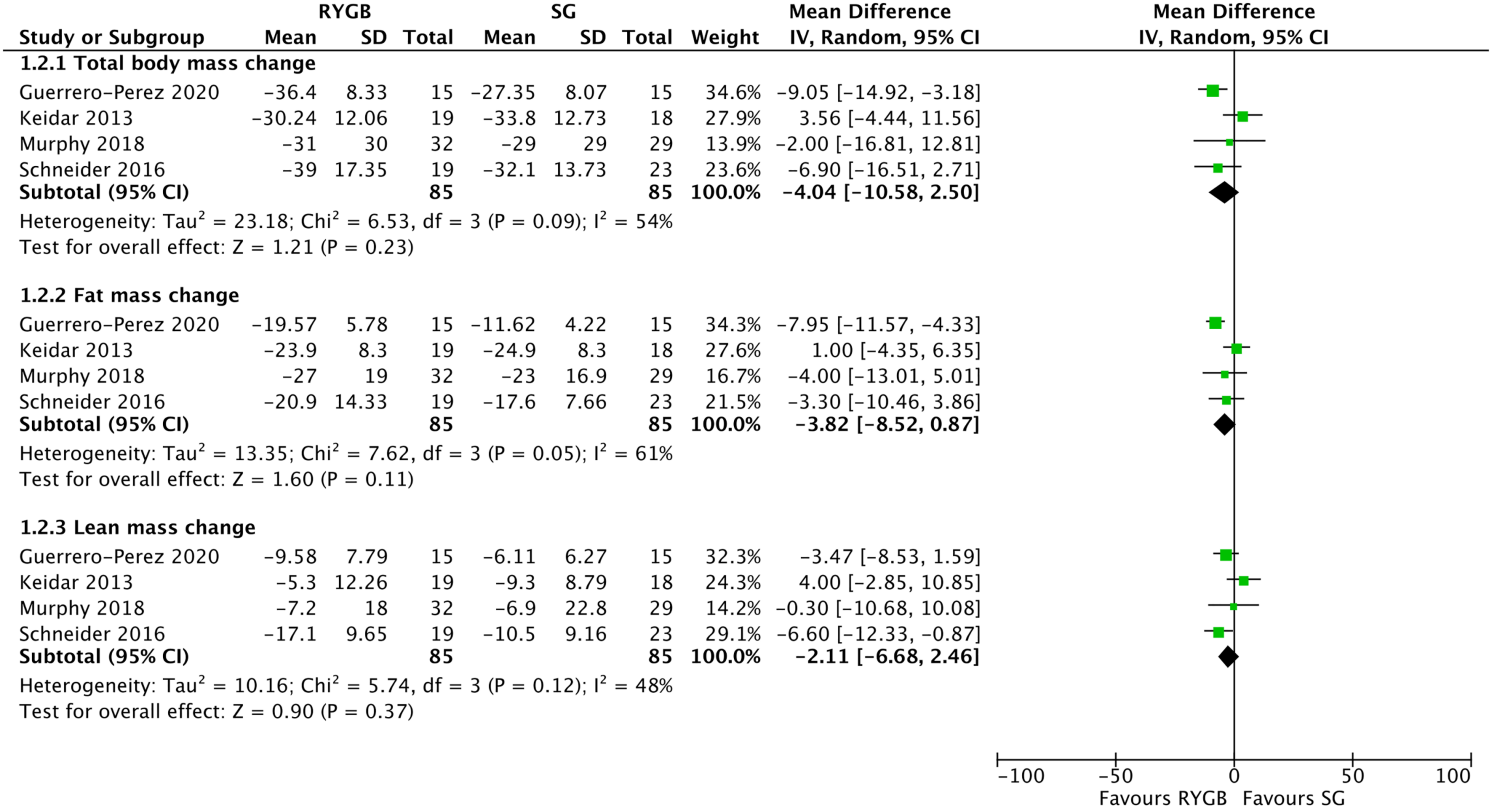
Mean differences in body composition outcomes after Roux‐en‐Y gastric bypass and sleeve gastrectomy

Four studies were eligible for inclusion in the RYGB versus SG meta‐analysis, with a total of 170 participants (Figure 4).^25^, ^26^, ^27^, ^40^ Total body mass, fat mass, and lean mass losses were similar in those who underwent RYGB compared with SG. However, heterogeneity was high for all comparisons. Of the studies, two^26^, ^40^ were identified as having some concerns of risk of bias. A sensitivity analysis for low risk of bias studies demonstrated that RYGB results in greater total body mass (MD: −8.47 kg [95% CI: −13.47, −3.46], *P* = 0.0009), fat mass (MD: −6.69 kg [95% CI: −10.74, −2.65], *P* = 0.001), and lean mass changes (MD: −4.84 kg [95% CI: −8.63, −1.05], *P* = 0.01) than SG. The two studies^26^, ^40^ which had some risk of bias were also the same two studies which reported fat‐free mass instead of lean mass results.

We performed additional sensitivity analyses where we substituted our imputed correlation coefficient of 0.7 with values of 0.5 and 0.9 and neither coefficient changed our overall findings.

## 4 DISCUSSION

RYGB results in greater total body and fat mass losses but similar lean mass losses compared with gastric banding and similar body composition changes compared with SG at 12 months. Although limited by the low number of studies, comparative to gastric banding, our results demonstrated that RYGB might lead to greater post‐surgery fat loss without greater lean mass loss.

### 4.1 RYGB versus gastric banding

Unfortunately, due to one of the two included studies having a moderate risk of bias, the generalizability of our findings for this meta‐analysis are limited. However, we propose two speculative mechanisms by which RYGB may result in greater fat mass and equivalent lean mass changes compared with gastric banding.

The greater fat mass losses observed following RYGB relative to gastric banding might be related to the greater increases in glucagon‐like peptide‐1 (GLP‐1) that occur following RYGB compared with gastric banding.^43^ GLP‐1 is known to increase post‐prandial insulin secretion and induce a sensation of satiety, thus making it a key factor for the regulation of glucose and appetite control.^44^ This is supported by the 1‐year post‐surgery outcomes of Holter et al.,^45^ which demonstrated that patients affected by obesity and T2DM who undergo RYGB have a higher insulin secretion rate within the first 60 min and lower glucose levels 120 min after ingesting glucose, compared with those who undergo gastric banding. In addition to decreasing appetite, high GLP‐1 levels may reduce an individual's preference for high‐calorie salty foods.^45^ Investigation into the mechanism of GLP‐1 agonists (which are used as novel therapy option for T2DM) further support the notion that GLP‐1 promotes weight loss through fat mass losses.^46^ A recent study demonstrated that adults with obesity who took the GLP‐1 agonist liraglutide for 16 weeks experienced significant android fat mass losses, but no lean mass losses, compared with placebo groups.^45^ Thus, higher GLP‐1 levels post‐RYGB compared with gastric banding may confer greater fat mass losses. Given the speculative nature of this theory, more research would be required to confirm these associations.

Changes in food preference might also explain why fat mass losses were greater in those who underwent RYGB relative to gastric banding. RYGB is associated with increased consumption of “healthy” foods (such as fruits, fish, and less processed foods),^47^, ^48^ with energy intake from fat consumption decreasing from 37.0% immediately before surgery to 25.3% 2 years post‐surgery (*P* < 0.0001).^48^ In comparison, after 1 year, the average energy intake of patients undergoing gastric banding continued to be 36% total fats.^49^ In both studies, patients were given similar dietary advice post‐surgery, but no specific diet was prescribed. Additionally, another study used the validated “Swedish Obese Subjects” questionnaire to calculate percentage of total energy intake for patients 1‐year post‐surgery and found that those who underwent gastric banding consumed higher amounts of sweets and fats compared with those who underwent RYGB.^41^ Conversely, energy from fruits and vegetables following RYGB was over 10% of daily total intake as compared with around 2.5% for those who underwent gastric banding (*P* < 0.0001). A recent systematic review^50^ analyzed the difference in food preferences post‐surgery. It determined that poorer diets post‐gastric banding compared with RYGB may be due to the purely restrictive nature of the procedure and minimal influence on metabolism of food, with gastric banding patients more likely to feel hungry and adopt a high‐calorie diet to compensate.^50^ Further research is required to understand changes in eating behavior and its influence on body composition changes in those who undergo RYGB compared with gastric banding.

### 4.2 RYGB versus SG

Our finding that RYGB resulted in similar body composition changes compared with SG is supported by multiple cohort studies comparing adults with obesity undergoing RYGB or SG.^51^, ^52^, ^53^ One study followed up participants at 6 months^51^ and another after 1 year,^52^ and the most recent was a long‐term review at 6.7 years post‐bariatric procedure.^53^ At each of these time‐points, there were no significant differences in body composition outcomes.^51^, ^52^, ^53^ Additionally, a recent meta‐analysis^54^ found body mass changes post‐surgery were sustained over 5 years for both RYGB and SG. Although our limited data meant that we could not explore long‐term body composition changes in our meta‐analysis, our findings suggest that the lean and fat mass changes post RYGB and SG are comparable at 12 months.

There is evidence that different initial body weight and body composition influences the partitioning of weight loss as either fat or lean mass, and effects of initial weight on weight loss and body composition changes were not explored in this study. However, given that only RCTs were included in the analysis and initial total body, fat and lean mass were similar between groups reduces the influence on body weight and body composition outcomes in our study. The Forbes theory equation is used to predict changes in fat‐free mass during weight change as a function of the initial body fat mass.^55^ However, this equation may only be valid for measuring moderate amounts of weight loss; for more extreme weight change as seen post‐bariatric surgery, this equation underestimates the proportion of fat‐free mass loss.^55^ Hall's theory may be a better predictor of relative loss of fat‐free mass versus fat mass following bariatric surgery,^56^ but there is no universally accepted equation for predicting change in fat‐free versus fat mass for people undergoing dramatic weight loss.

As seen in amended Table 1, baseline body composition was very similar between groups that were compared. We have also presented the mean total body, fat and lean mass at baseline in each surgical group in our results text, which were also very similar between different surgical groups.

### 4.3 Limitations

There were several limitations to our analysis. First, we only had access to aggregate data, not individual patient data. The low number of included studies could have led to some of our analyses being under‐powered, resulting in an inability to detect significant differences between groups. Furthermore, we were unable to conduct several subanalyses including younger versus older participants and different comorbid diseases. The inclusion of cohorts with comorbidities such as T2DM might have influenced body composition changes in response to different surgical weight loss interventions, thereby limiting the generalizability of our findings. Postoperative interventions (e.g., prescription of daily oral supplements such as vitamin D, multivitamin, calcium, and non‐tailored behavioral weight control interventions such as in‐person group workshops) were heterogenous and it was not possible to compare effects these on weight loss and body composition changes. Furthermore, no included studies prescribed a specific dietary and or exercise lifestyle post operative interventions. We were therefore unable to determine whether observed differences are wholly attributable to procedure type or in part related to postoperative management. We were unable to assess publication bias due to the limited studies available for the analysis. Due to the limited number of RCT available assessing body composition changes after different bariatric procedures, we also were unable to compare all types of surgery in current practice (e.g. anastomosis gastric bypass and biliopancreatic diversion with duodenal switch). Therefore, our conclusions are limited to the procedures presented in our analyses rather than an overview of all procedures in current practice.

Regarding measurement tools used to assess body composition, we were unable to perform an additional analyses to compare effects of use of DXA versus BIA to quantify body composition due to the low number of included studies. BIA uses electrical currents to determine conductivity of different tissue and equations to calculate the proportion of body fat based on the assumption of a constantly hydrated body.^57^ In contrast, DXA uses X‐ray attenuation by different tissues to estimate body composition and its accuracy can be influence by body size.^58^ Therefore, in populations with obesity, differences in total body water levels and body composition (e.g., greater tissue depth) may decrease the accuracy of body fat and fat‐free mass estimates.^57^ There were two types of DXA machines (GE/Lunar and Hologic) that were utilized by our included studies, and while DXA is not considered a gold‐standard measurement for body composition, it is widely recommend due to its high precision, low cost, and wide availability.^59^ Each type has different degrees of precision due to proprietary calibration methods and equations for calculation of body composition results^60^; however, we were unable to stratify by machine type due to low number of studies. Other DXA limitations in populations with obesity also include machine weight limits (~136–156 kg) and scan area restrictions (~195 cm high and ~67 cm wide) for machines used in included studies.^61^ However, given the average body mass was <115 kg in all groups included in this meta‐analysis and that right side half‐body analysis in obese subjects appears to be very comparable with whole‐body analysis for measuring body composition (>99.9% in 52 subjects with a BMI > 30 kg/m^2^),^62^ it is unlikely these limitations had significant effects on outcome measures reported in this study. These limitations for both DXA and BIA are further accentuated during periods of weight fluctuation as non‐steady state body weight conditions influence accuracy of estimates for body composition.

Ultimately, it is unclear what constitutes a clinically meaningful loss of lean mass, and this is difficult to ascertain in the context of bariatric surgery. Indeed, it is likely that any detrimental effects of loss of lean mass on function and metabolic health in bariatric surgery are offset by the overwhelming benefits associated with significant loss of fat mass. However, it is possible that substantial losses of lean mass “blunt” the positive effects of fat loss, and further research is required to determine relative changes in lean mass and its association with functional and metabolic outcomes post‐operatively.

This meta‐analysis demonstrated that RYGB leads to greater total body and fat mass losses, but similar lean mass losses, compared with gastric banding at 12 months. Body composition changes following RYGB relative to SG were similar. The limited studies available for this analysis highlight the necessity for further RCTs comparing body composition changes following different bariatric surgery procedures. In particular, additional studies of durations beyond 12 months are required to determine whether maintenance of relative muscle mass influences long‐term functional and metabolic outcomes following bariatric surgery in adults with obesity.

## CONFLICT OF INTEREST

The authors declare no conflict of interest.

## AUTHOR CONTRIBUTIONS

AS and PJ drafted the manuscript. PJ, AS, JM, and DS were responsible for manuscript revision and preparation of this review. All authors have read and approved the manuscript.

## Supporting information




**Table S1:** Prisma Checklist (2020)
**Table S2:** Search strategy (Web of Science)
